# Application of Calcium Phosphate Materials in Dentistry

**DOI:** 10.1155/2013/876132

**Published:** 2013-06-26

**Authors:** Jabr S. Al-Sanabani, Ahmed A. Madfa, Fadhel A. Al-Sanabani

**Affiliations:** ^1^Department of Oral Medicine and Oral Diagnosis, Faculty of Dentistry, University of Thamar, Dhamar 87407, Yemen; ^2^Department of Conservative Dentistry, Faculty of Dentistry, University of Thamar, Dhamar 87407, Yemen

## Abstract

Calcium phosphate materials are similar to bone in composition and in having bioactive and osteoconductive properties. Calcium phosphate materials in different forms, as cements, composites, and coatings, are used in many medical and dental applications. This paper reviews the applications of these materials in dentistry. It presents a brief history, dental applications, and methods for improving their mechanical properties. Notable research is highlighted regarding (1) application of calcium phosphate into various fields in dentistry; (2) improving mechanical properties of calcium phosphate; (3) biomimetic process and functionally graded materials. This paper deals with most common types of the calcium phosphate materials such as hydroxyapatite and tricalcium phosphate which are currently used in dental and medical fields.

## 1. Introduction 

Calcium phosphate materials have received a lot of research attention in recent years due to their chemical similarity to bones and teeth. They are attractive biomedical materials owing to their excellent biocompatibility and the nontoxicity of their chemical components [1–4]. 

Calcium phosphates belong to the group of bioactive synthetic materials and its most frequently used are the hydroxyapatite and the tricalcium phosphate. These types are commonly used due to their osteoconductivity, crystallographic structures, and chemical composition similar to the skeletal tissue. They are classified according to their “resorbability,” that is extent of degradation *in vivo*. Hydroxyapatite has been described as “nonresorbable” and tricalcium phosphate has been described as “resorbable” [3, 4]. 

Calcium phosphate materials show a positive interaction with living tissue that includes also differentiation of immature cells towards bone cells [4, 5]. These materials also have chemical bonding to the bone along the interface, thought to be triggered by the adsorption of bone growth-mediating proteins at the biomaterials surface [4, 6]. Hence, there will be a biochemically mediated strong bonding osteogenesis [6, 7]. In addition to compressive forces, to some degree tensile and shear forces can also be transmitted through the interface (“bony ingrowth”). 

The first calcium phosphate materials were used in the 1920s. They were used as bone substitute or bone graft [8]. It was reported that a “triple calcium phosphate” compound used in a bony defect promoted osteogenesis or new bone formation. In 1971, Monroe and his colleagues reported a method for the preparation of a calcium phosphate, principally mineral calcium-fluorapatite, and suggested the possible use of this apatite ceramic for dental and medical implant materials [9]. The first dental application was reported by Nery et al. [10] more than many years later using a synthetic porous material obtained by sintering a “tricalcium phosphate reagent” that was originally described by the authors as “tricalcium phosphate” but later demonstrated to consist of a mixture of hydroxyapatite and tricalcium phosphate [11].

Applications of calcium phosphates include repair of periodontal defects, augmentation of alveolar bone, sinus lifts, tooth replacement, and repair of large bone defects caused by tumors [12–18]. They are also used as scaffolds in tissue engineering for bone or dentin regeneration [18–22]. Calcium phosphates are also used in the form of injectable cements [23, 24] or as coatings on titanium and titanium alloy implants to combine the bioactivity of the calcium phosphates and the strength of the metal [25, 26]. 

The purpose of the present paper is to review the use of calcium phosphate materials in dentistry. Emphasis will be given to the hydroxyapatite and tricalcium phosphate. This review summarizes brief history, dental applications, and methods for improving their properties.

## 2. Hydroxyapatite

### 2.1. Overview

Hydroxyapatite is the most documented calcium phosphate and can be used in bulk form, as a coating and/or cements [27–29]. This material can be classified according to its porosity, phase, and processing method. It has excellent biocompatibility and is able to promote osteoconduction and osseointegration. As a result of excellent favorable osteoconductive and bioactive properties, it is widely preferred as the biomaterial of choice in both dentistry and orthopedics [30–32].

### 2.2. Composition and Structure of Hydroxyapatite

Synthetic hydroxyapatite is similar in composition to the mineral component of bone and teeth as shown in Table 1 [33]. This similarity makes it the most clinically used as biomaterial for medical and dental applications [34]. 

Hydroxyapatite has a hexagonal symmetry and unit cell lattice parameters *a* = 0.94 nm and *c* = 0.68 nm. Taking into account the lattice parameters and its symmetry, its unit cell is considered to be arranged along the *c*-axis. This would justify a preferred orientation that gives rise to an oriented growth along the *c*-axis and a needle-like morphology.  Table 2 shows the similarities in crystallographic properties: lattice parameters (±0.003 Å) between enamel, dentine, bone, and hydroxyapatite as reported by Dorozhkin [33].

### 2.3. Properties of Hydroxyapatite

Although hydroxyapatite has favorable bioactive and osteoconductive properties that result in rapid bone formation in a host body and strong biological fixation to bony tissues [35], it possesses low mechanical strength and fracture toughness, which is an obstacle to its applications in load-bearing areas [36]. Typical properties of dense hydroxyapatite are given in Table 3 [37]. Thus, the enhancement of the mechanical properties of hydroxyapatite would extend its scope of applications.

### 2.4. Application of Hydroxyapatite

Hydroxyapatite has been used successfully in clinical and animal studies for endodontic treatment including pulp capping, repair of mechanical bifurcation perforation, apical barrier formation, and repair of periapical defects [38–41]. Jean et al. [38] suggested that the degree of mineralization of reparative dentin formation obtained with tricalcium phosphate-hydroxyapatite was quicker and thicker when compared with that produced by calcium hydroxide. Additionally, hydroxyapatite has been used as filler for reinforcing dental resins [42, 43], coating in both orthopedic and dental implants [44, 45], restoration of edentulous atrophic ridges [46], intrabony periodontal pockets (a periodontal pocket in which the bottom is apical to the level of the adjacent alveolar bone) [47], periodontal defects [48], under and around failing subperiosteal metal implants [49], and ridge augmentation prior to implant for metal prosthetics [50].

### 2.5. Improving in the Properties of Hydroxyapatite

#### 2.5.1. Hydroxyapatite Composites

Combinations of hydroxyapatite with synthetic polymers or metallic agents are called hydroxyapatite composites. They have been developed and studied in purpose to improve the mechanical properties of porous hydroxyapatite, or vice versa, to develop partially biodegradable artificial bone grafts for tissue engineering [32, 51]. 

A number of reinforcements, including particles, platelets, whiskers, long fibers, partially stabilized zirconia, metal dispersoids, and polymers, have been used in hydroxyapatite to improve their reliability [52–55]. Deng et al. [56] added nanocrystalline hydroxyapatite to a polylactide solution to form solvent-cast composite matrices and found a steady increase in tensile modulus as hydroxyapatite loading increased from a low of 1.66 GPa for polymer without hydroxyapatite up to 2.47 GPa for 10.5% hydroxyapatite content. Wang et al. [57] combined polyamide, a bioinert polymer, with both microcrystalline and nanocrystalline hydroxyapatite and compared the resulting bending strength and tensile strength. As the ceramic content of each composite increased, so did the bending strength. For both bending and tensile strengths, the addition of nanocrystalline hydroxyapatite increased the properties over those with microcrystalline hydroxyapatite. It was theorized that the smaller crystals of the nanocrystalline hydroxyapatite resulted in higher surface areas and thus greater surface energy, surface activity, and thus bonding between the polymer and the hydroxyapatite. Abu Bakar et al. [58] examined the effect of varying amounts of hydroxyapatite added to polyetheretherketone as an injection-molded composite by varying hydroxyapatite content between 0 and 40% by volume. Results indicate that Young's modulus increased from approximately 3 to 15 GPa as hydroxyapatite content increased from 0 to 40% but tensile strength decreased from 80 to 44 MPa along the same increase in hydroxyapatite content. Balać et al. [59] attempted to understand the effect of hydroxyapatite particle shape and volume fraction on a polylactide/collagen/hydroxyapatite composite scaffold using finite element analysis and found fewer stress concentrations throughout the matrix with an increased hydroxyapatite volume fraction but a reduced dependence on this as the hydroxyapatite particles were modeled as spherical, suggesting yet another design consideration for the composite scaffold. Hydroxyapatite matrix composites containing 20–30% Fe-Cr alloy long metal fibers showed the highest values of fracture toughness and fracture strength for hydroxyapatite based materials as reported by Suchanek and Yoshimura [60]. Ramires et al. [61] tested titanium oxide and hydroxyapatite composites, formed by sol-gel method, for biocompatibility and cell response. Their results showed that the combination was biocompatible and excellent at promoting cell activity. Volceanov et al. [62] investigated the influence of zirconia addition to a hydroxyapatite matrix on mechanical strengths and the interaction mechanism between zirconia and its polymorphs with calcium phosphates after sintering at 1250°C. Their results highlighted that there were improved mechanical properties for hydroxyapatite matrix composites cured in air at 1250°C. Some authors added small amounts of P-glass into hydroxyapatite to improve sinterability and mechanical properties of the dense body, as well as biological properties [63–65]. Ferraz et al. [63] fabricated glass-hydroxyapatite composite coatings, using a plasma spraying technique, and experimented it *in vitro *with osteosarcoma cells. Their findings showed favorable cellular responses. Others claimed that the inclusion of phosphate-based glasses produced significant improvement in mechanical properties [66, 67].

## 3. Hydroxyapatite Coatings

### 3.1. Overview

Bioactive calcium phosphate ceramics as coatings on bioinert metallic substrate have received worldwide attention in both orthopaedic and dental implants due to their biocompatibility and their ability to bond directly to bone [68]. However, there are several factors that may influence the performance of any hydroxyapatite coating such as coating thickness, chemical composition, crystallinity, phase purity, cohesive and adhesive strengths, and resorption resistance. Adhesion strength of the coating to the implant surface appears to be a property that needs to be maximized to avoid cracking, shearing off, and chipping of the hydroxyapatite coating during emplacement of the implant. The ideal hydroxyapatite coating would be one with low porosity, strong cohesive strength, good adhesion to the substrate, a high degree of crystallinity, high chemical purity, and phase stability [69]. 

### 3.2. Stability of Hydroxyapatite Coatings

Many studies have indicated that the dissolution of well-crystallized hydroxyapatite in the human body after implantation is too low to achieve optimum results [70–72]. On the other hand, the dissolution rate of tricalcium phosphate is too fast for bone bonding. To achieve an optimum dissolution rate of bone graft materials, research has focused mainly on biphasic calcium phosphate ceramics composed of hydroxyapatite and tricalcium phosphate [73, 74]. It is generally known that tricalcium phosphate is more soluble than hydroxyapatite at physiologic pH and more susceptible to bioresorption [75]. Partial dissolution of the calcium phosphate macrocrystals followed by an increase in the calcium and phosphate ion concentrations in the local environment is thought to be important for the excellent osteoconductivity and tight chemical bonding of the bioactive ceramics with bone [76]. Although greater, unpredictable solubility of the tricalcium phosphate coating may cause earlier failure of a hydroxyapatite/tricalcium phosphate-coated implant at the bone-implant interface [77], gradual resorption of this coating and replacement with new bone might be desirable to prevent the late complications of calcium phosphate coatings [78]. 

### 3.3. Applications of Hydroxyapatite Coatings

In the 1960s, the concept of biological fixation of load-bearing implants using bioactive hydroxyapatite and calcium phosphate coatings was proposed as an alternative to cemented fixation. Since Furlong and Osborn first began clinical trials using the hydroxyapatite-coated implants in 1985 [79], it has been reported that hydroxyapatite coatings can successfully enhance clinical success, and a less than 2% failure rate was reported during a mean follow-up study of 10 years [80, 81]. Hydroxyapatite is stable in a body fluid, whereas tricalcium phosphate is rather soluble in the fluid [82]. 

### 3.4. Osseointegration Hydroxyapatite-Coated Dental Implants

Since the clinical success of orthopaedic and dental implants depend on the osseointegration at the bone-implant interface, surfaces of bone-contacting devices would be desirable to be compositionally, structurally, and functionally analogous to that of human bone. Surface composition containing calcium and phosphate displays good cytocompatibility and enhanced bone contact and greater new bone apposition, particularly calcium. Okamoto et al. [83] reported that a significantly higher number of cells adhered to hydroxyapatite than to uncoated titanium. Wong et al. [84] compared the osseointegration of commercial implants in the trabecular bone of mature miniature pigs for 12 weeks. Their results showed excellent osseointegration of the hydroxyapatite coated implant. Likewise, Cao et al. [85] showed successful osseointegration of hydroxyapatite coatings with surrounding bone tissue when a hydroxyapatite coated implant was placed within living bone. Also, the success or failure of hydroxyapatite coated orthopaedic implants depends on the control and consequences of cell behaviour after implantation [86]. Thus, the first and essential step for bone tissue-implant interface studies is *in vivo* tests using osteoblast cells due to the important role which they play in the osteointegration of the implant. They have the ability to synthesise and produce extracellular matrix and to control its mineralization and thus regulate the “ingrowth” of bone to the implant. Rouahi et al. [87] examined the growth of Saos-2 cells on discs of microporous and nonporous hydroxyapatite in comparison to titanium. The surface morphology was found to have an effect on the behavior of the cells. Richard et al. [86] cultured cells on calcium-deficient hydroxyapatite thin films produced using electrodeposition. Areas of the coating with two different morphologies and compositions were examined, and the results were compared to those for cells cultured on cell culture plastic. In this study, cell morphology, cell viability, cell proliferation, and gene expression were examined over 28 days. The differentiation of osteoblast cells was found to be enhanced on the calcium phosphate coating compared to the titanium plate. Yang et al. [88] reported that cell proliferation and type I collagen synthesis were higher on porous surfaces than on dense ones. This is related to greater protein absorption and to the increased surface area available for cell attachment. Wang et al. [89] carried out a study to determine the effect of the phase composition of calcium phosphate ceramics on osteoblast behaviour. The compositions studied were pure hydroxyapatite, a 70/30 mixture of hydroxyapatite and tricalcium phosphate, and a 35/65 mixture of hydroxyapatite and tricalcium phosphate and pure tricalcium phosphate. In their study, the phase composition of the ceramics did not have a significant affect on the expression of the osteonectin and production of bone sialoprotein and osteocalcin in SaOS-2 cells. 

Histologically, comparing osseous apposition to hydroxyapatite coated implants and titanium implants has demonstrated mineralization of bone directly on hydroxyapatite surfaces with no fibrous tissue layer formation. However, a predominately fibrous tissue interface was observed on titanium implants, with only minimal areas of direct bone contact [90]. In addition, in an animal study hydroxyapatite-coated implants showed an increased coronal bone growth that was not observed with titanium implants [91]. Maintaining a bony osseous crest is clinically essential because it may prevent peri-implant saucerization and subsequent pocket formation [92, 93]. Other histometric studies in animal models have also exemplified that bone adapts in much less time to hydroxyapatite-coated implants than to titanium implants [94, 95].

### 3.5. Recent Advance in Utilization of Hydroxyapatite Coatings

Another area of recent advance is the use of drug-releasing layers on hydroxyapatite coatings. These layers are designed to supply drugs, for example, antibiotics and antiresorptive drugs, locally to the bone surrounding the implant. Drug-releasing layers have been produced from numerous different polymeric and ceramic materials. The benefits of these drug-releasing coating layers have been shown by a number of researchers [96, 97]. Ogiso et al. [96] used the antiresorptive drug zoledronate grafted to a hydroxyapatite coated implant. *In vivo* studies in rats showed an increase in mechanical fixation of the implants. Martins et al. [97] found that their collagen-hydroxyapatite composite paste had potential for use in sustained antibiotic release.

## 4. Tricalcium Phosphate

### 4.1. Overview

Tricalcium phosphate exists in many polymorphs (*α*, *β*, *γ*, and super-*α*) [98]. The only two polymorphs phases (*α* and *β*) are used as biomaterials. These phases have received much attention [99]. However, despite the extensive research since the early 1970s, there is still lack of clarity concerning this material. The use of resorbable tricalcium phosphate materials is preferred since they will be in the long term replaced by bone. 

### 4.2. Application of Tricalcium Phosphate

Tricalcium phosphate materials mostly behave as osteoconductive materials, which permits bone growth on their surface or into pores, channels, or pipes [100]. Calcium phosphate is biocompatible material and useful for inducing hard tissue formation [101, 102]. It has been used as capping agent [101], cleft palate [103], apical barrier [104], apexification [105], vertical bone defect [106], and implants coating [75]. Tricalcium phosphate is a resorbable phase calcium phosphate and exhibits some good properties. It has also been shown to support bone growth [107]. However, it is difficult to sinter, showing poor mechanical strength and low resistance to crack-growth propagation. Further, the rate of resorption of tricalcium phosphate is fast and uncontrolled [108]. Unpredictable solubility of the tricalcium phosphate coating may cause earlier failure of coated implant.

Clarke et al. [109] reported a method of preparing tricalcium phosphate ceramic and suggested its use as a bone graft material. Levin et al. [110] reported that the first dental application of a tricalcium phosphate ceramic in periodontal defects in dogs. Koenigs et al. [111] used resorbable form of tricalcium phosphate ceramic to induce apical closure. Formation of mineralized tissue occurred within the root canal, but was incomplete. Roberts and Brilliant [112] used tricalcilum phosphate ceramic to induce apical closure in human permanent pulpless teeth with large open apices, but found it to be no more effective than calcium hydroxide. Brown and Chow [113] tested a tricalcium phosphate and brushite combination. X-ray diffraction revealed a conversion to HA in a few minutes with compressive strengths of up to 500 psi. Gruninger et al. [114] tested the apical barrier of 101 teeth. They found that no difference in healing between cases treated with tricalcium phosphate or calcium hydroxide. Wong et al. [115] tested a combination of tricalcium phosphate, hydroxyapatite, and sodium fluoride as a bone implant material. They determined the material to be neither toxic nor mutagenic and not resorbable. They encouraged the evaluation of these materials as root canal filler. Functionally graded coatings consisting of fluorine-substituted apatite (FA) and beta-tricalcium phosphate (*β*-TCP) were also produced by Takechi et al. [116]. The coating produced had four layers: the outermost layer containing FA + 50 wt% TCP, the next FA + 40 wt% TCP, + 30 wt% TCP, and finally the innermost FA + 20 wt% TCP. The HA component of the coating is expected to enhance early-stage bone ingrowth and bone bonding, whereas the remaining porous FA component is aimed at achieving long-term fixation of an implant.

## 5. Calcium Phosphate Cement Systems

### 5.1. Overview

Calcium phosphate cement is bioactive cement that sets as hydroxyapatite when moistened [117]. Calcium phosphate cement was discovered by Brown and Chow in the 1980s. This type of cement can be prepared by mixing a calcium phosphate salt with water or with an aqueous solution to form a paste that reacts at room or body temperature, giving rise to a precipitate containing one or more calcium phosphates, which sets by the intercrossing of the crystals of this precipitate. This cement consists of two components, one basic and one acid, which react when mixed with water, producing one or more products with an intermediary acidity [118, 119]. 

In 1998, Böstman [120] presented preliminary studies on the possibility of developing apatitic calcium phosphate cements, with the rationale that such cements would have the unique combination of the following properties: (i) compatibility with the tooth mineral; (ii) adjustability of composition (with or without F~, Mg^2+^, Sr^2+^, etc.); and (iii) esthetics.

### 5.2. Application of Calcium Phosphate Cements

Calcium phosphate cements have been evaluated as one of the potential materials for bone tissue engineering. An advantage of calcium phosphate cement is that they can be directly injected into the bone defect and allowed to set *in situ*. Calcium phosphate cements also are biocompatible and resorbable; they can be synthesized with a macroporous structure having micropores that are very crucial for cellular growth and infiltration [121, 122]. In 2002, Mickiewicz et al. developed a novel class of low-temperature setting calcium phosphate cements from precursors such as dicalcium phosphate dihydrate, dicalcium phosphate anhydrous, and tetracalcium phosphate [123]. These low-temperature apatites are receiving a great deal of attention due to their ability to set at physiological temperature to form hydroxyapatite that resembles biological apatites without the addition of any additives [124, 125]. This is highly advantageous because acrylic cements currently used for orthopedic applications require high temperature for setting and use of toxic reagents [124]. Another advantage of calcium phosphate cement is that during the setting reaction only a small amount of heat is released as compared to polymethylmethacrylate cements and also the volume of calcium phosphate cement remains constant during the setting reaction [124].

Upon mixing with water or aqueous solution, the calcium phosphate cement dissolves and precipitates into a less soluble calcium phosphate. During precipitation, the calcium phosphate crystals increase in size and gets interlocked, thus providing structural rigidity to the cement. Hydroxyapatite thus formed in aqueous solution is poorly crystalline [124]. When used for *in vivo* applications, a thick paste of calcium phosphate cement can be formed in the presence of water or aqueous solutions which can be injected or sculpted during surgery into the defect site and self-hardens to form hydroxyapatite *in situ* [125–127]. Hence, these biomaterials do not require shaping and can be prepared at operating room conditions. They provide excellent contact between the bone and the graft. Since most of the current orthopedic implants are available in hardened form, the moldability and *in situ* hardening of calcium phosphate cement along with its osteocompatibility make it a desirable alternative for current orthopedic implants. Moreover, since the calcium phosphate cements are fabricated at room or at body temperatures, also they can be used as drug delivery vehicle for antibiotics, antitumor drugs, anti-inflammatory drugs, and growth factors [128, 129]. However, currently available calcium phosphate cement systems are far from ideal properties due to the discrepancies in the setting time, mechanical properties, and *in vivo *response of the cements [130]. Also, they are used under development for furcation sealing, root surface desensitization, and root apex sealing or root canal filling [131, 132]. The abilities of self-setting, fair compressive strength, and biocompatibility suggest that calcium phosphate appears superior to pure calcium hydroxide; thus, this material may have potential for dentine regenerating pulp capping or lining materials [133, 134]. Calcium phosphate cement systems also have been used as bone fillers and to deliver bioactive agents due to their osteoconductivity, osteotransductivity, and suitable mechanical properties [131–138].

## 6. Future Opportunities in Calcium Phosphate Applications

### 6.1. Biomimetic Process

Some authors reported deposition of long and thin needle-shaped crystals of enamel-like calcium phosphate onto a bioactive glass in a supersaturated calcifying solution containing recombinant porcine amelogenins [139, 140]. It has been realized that nucleation and growth of calcium phosphate crystals *in vivo* are modulated by specific proteins in mineralizing tissues, intrinsically by functional groups in proteins. Other authors reported that some functional groups have the ability to induce bone-like apatite nucleation through a biomimetic way [140, 141]. A self-assembled monolayer (SAM) technique is an effective way to fabricate charged surface terminated with polar head groups [142]. The deposition of bone-like apatite could improve the biological properties for potential restorative application. Thus, biomimetic strategies developed to design new materials, which are expected to improve biological and mechanical performance for biomaterials [143, 144]. 

Many researchers used bovine and human sera *in vitro* to analyze protein adsorption on biomaterials [145, 146]. The reactions occurring at the surface of biomaterials in contact with protein containing solutions have also been studied with Dulbecco's Modified Eagle's minimum essential medium supplemented with 10% Nu-Serum [147], which contains growth factors, hormones and vitamins. A step further to simulate *in vitro* the real condition of biomaterials immersed into body fluids is the immersion in cell-containing solutions. 

### 6.2. Functionally Graded Materials

Functionally graded materials are a group of new materials that have recently attracted much attention. They are advanced composite materials that are engineered to have a smooth spatial variation of material properties. This is achieved by fabricating the composite material to have a gradual spatial variation of the relative volume fractions and microstructure of its material constituents. The choice of material phases is motivated by functional performance requirements. Therefore, functionally graded materials permit tailoring of material composition so as to derive maximum benefits from their inhomogeneity [148]. A functionally graded material is obtained by varying the composition from one side of the material to the other side either continuously or stepwise. This graded structure allows the integration of dissimilar materials such as ceramics and metals without severe internal stress and combines diverse properties into a single material system. Thus, it can perform specific functions and meet performance requirements [149].

According to Narayan et al. [150], functionally graded material was proposed in 1984 at the National Aerospace Laboratory of Japan by Niino and his coworkers, as a preparation method for thermal barrier material for space plane application. This concept has been later expanded for different applications such as coatings, packing, optics, biomedics. In the biomedical filed, several approaches have been used to develop functionally graded biomaterials for implants [151]. Functionally graded materials can be made into bulky specimens with strong interfacial bonding and reduced thermal stresses unlike coatings on substrates. As a biomaterial, a bulky hydroxyapatite/titanium functionally graded composite was prepared by a powder metallurgy method [152]. 

Chu et al. [153] tested the bending strength of hydroxyapatite/titanium functionally graded implant-bone-bond to be 159 MPa. Zhu et al. [154] tested the bonding shear strength of hydroxyapatite/titanium functionally graded implant to be 6.49 MPa after being implanted for 3 months. Chu et al. [155] designed optimally and fabricated hydroxyapatite/titanium functionally graded material, based on the criterion of minimum residual thermal stress. The titanium component improved the mechanical properties of the coating and also assisted in reducing the residual stresses in the final coating, as the thermal expansion coefficient was gradually increased from the substrate to the outer layer of the coating. Khor et al. [148] also produced hydroxyapatite-titanium functionally graded coatings. This research used the titanium alloy, Ti-6Al-4V and found improvements in microstructure, density, porosity, microhardness, and Young's modulus. Hedia and Mahmoud [156] used the finite-element method to optimize the hydroxyapatite/titanium functionally graded dental implant, based on the criterion of minimum von Mises' stress. Hedia [157] later improved the analysis by including this effect in another numerical investigation. Yang and Xiang [158] investigated the biomechanical behaviour of a threaded functionally graded biomaterials dental implant/surrounding bone system under static and harmonic occlusal forces by using a three-dimensional finite-element method. They concluded that functionally graded biomaterials dental implant effectively reduces the stress difference at the implant-bone interfaces where maximum stresses occur. Also, Wang et al. [159] investigated the thermal-mechanical performance of hydroxyapatite/titanium functionally graded dental implants with the three-dimensional finite-element method. They concluded that, under the occlusal force only, the functionally graded implants with different hydroxyapatite fraction perform almost equally well, while the titanium yields much higher von Mises' stress. Functionally graded coatings containing hydroxyapatite and glass also were prepared by Yamada et al. [160]. The concentration of glass increased from the innermost to the outermost. The glass phase was found to improve adhesion of the coating to the titanium substrate.

The importance of the functionally graded material concept in biological applications and functions was reported by several studies [161, 162]. Fundamentally, the combination of mechanical properties and biocompatibility are very important factors in the application of any biomaterial to medical or dental field. The characteristics of the surface govern the biocompatibility of the material, and the mechanical strength is determined by the average mechanical strength of the materials. According to Chenglin et al. [163] and Lim et al. [164], the combinations of hydroxyapatite and Ti-6Al-4V can form an excellent functionally graded material. Since the surface layer is hydroxyapatite, the resultant functionally graded material shows excellent biocompatibility and bone-bonding ability or dental-bonding ability. Excellent mechanical strength in the functionally graded material is contributed by Ti-6Al-4V phase. Watari et al. [165] fabricated the hydroxyapatite/titanium functionally graded dental implant and tested its biocompatibility in Wistar strain rat. They observed that hydroxyapatite/titanium functionally graded dental implant had better biocompatibility than titanium implant. Yokoyama et al. [166] investigated the mechanical properties and biocompatibility of hydroxyapatite/titanium functionally graded implant fabricated by spark sintering method and reported that much improvement was achieved by this method. Miyao et al. [167] fabricated titanium/hydroxyapatite functionally graded material by spark plasma sintering, and the mechanical properties and biocompatibility as an implant were evaluated. He and his colleagues showed that the titanium/hydroxyapatite functionally graded material implants made by the spark plasma sintering method had strength, excellent biocompatibility, and controllability for graded bio-reaction. Foppiano et al. [168] evaluated *in vitro* the biocompatibility of functionally graded bioactive coating of novel glasses using mouse osteoblast-like cells. Their result exhibited that functionally graded bioactive coating performed at least as well as tissue culture polystyrene and Ti-6Al-4V alloy in the biocompatibility tests performed. In addition, functionally graded bioactive coating may affect gene expression favourably for osseointegration. Animal implantation tests have shown that the coexistence of the hydroxyapatite component in both the titanium/hydroxyapatite implants and bone accelerates the formation of new bone from earlier stage without inflammation [169]. Hedia [170] introduced the optimal design of functionally graded material dental implant in the presence of cancellous bone as a thin layer around the implant. The results exhibited that the optimal design of collagen/hydroxyapatite functionally graded material implant reduced the stresses concentration in the cortical bone, cancellous bone, and implant compared with conventional titanium. He also showed that collagen/hydroxyapatite functionally graded material had excellent biocompatibility and controllability. He claimed that the use of functionally graded material concept in dental implant materials achieve full integration of the implant with living bone, thus increasing the life of implant.

Huang et al. [171] added bioinspired FGM layer between the dental ceramic and the dental cement and investigated the effects of the functionally graded layer on the stress in the crown and its surrounding structures. From their results, the functionally graded layer was shown to promote significant stress reduction and improvements in the critical crack size. From their study, they concluded that the low stress concentrations were associated with the graded distributions in the dentin-enamel junction (DEJ). This provided new insights into the design of functionally graded crown architecture that can increase the durability of future dental restorations. Rahbar and Soboyejo [172] used computational and experimental effort to develop crack-resistant multilayered crowns that are inspired by the functionally graded DEJ structure. The computed stress distributions showed that the highest stress was concentrated at the ceramic outer layer of crown and reduced significantly towards the DEJ when bioinspired functionally graded architecture was used. They reported that the bioinspired functionally graded layers were also shown to promote improvements in the critical crack size.

Recently, Abu Kasim et al. [173] patented three types of multilayered composite materials that were produced using powders of zirconia (ZrO_2_), alumina (Al_2_O_3_), hydroxyapatite (HA), and titanium (Ti). The choice of powder for each layer was in accordance to the results of finite-element analysis 

Abu Kasim et al. [174] investigated the stress distribution of a newly designed functionally graded dental post (FGDP) which consisted of multilayer design of ZrO_2_-Ti-HA and compared to posts fabricated from homogeneous material such as titanium and zirconia. They concluded that FGDP exhibited several advantages in terms of stress distribution compared to posts fabricated from homogeneous material. The stress and strain distribution at the post-dentine interface of FGDP was better than that of homogenous posts. Therefore, it is important to ascertain the thermal behavior of FGDP in order to predict their performance in the oral environment.

## 7. Conclusions

This review article deals with some of the calcium phosphate materials which are currently used in dentistry and other calcium phosphate materials which have potential for dental applications. Although, calcium phosphate materials excellent bioactive and osteoconductive properties that results in rapid bone formation in a host body and strong biological fixation to bony tissues, calcium phosphate materials possess low mechanical strength, which is an obstacle to its applications in load bearing areas. Thus, the enhancement of the mechanical properties of calcium phosphate materials would extend its scope of applications. Calcium phosphate materials are either used as a bioactive coating on implants or reinforced with tough phases such as metal or ceramic phases for biomedical/dental applications.

This review also focuses on the development and current status of the calcium phosphate materials that were based on recent reviews. Methods for the development the properties of the calcium phosphate materials were based on data reported in the literature and on other studies by the authors.

New methods for fabrication the calcium phosphate materials developed to design new materials are also reviewed. The new trend for improving the mechanical and biological properties is biomimetic way and/or used functionally graded concept. These new trends in fabrication of calcium phosphate materials are expected to improve biological and mechanical performance for calcium phosphate materials.

Recent progress in the improving in biological and mechanical properties of calcium phosphate materials is reviewed. Although there was major improvement in the mechanical properties of calcium phosphate materials for dental and medical applications, further studies are needed to confirm their properties.

## Figures and Tables

**Table 1:** Chemical and structural comparison of teeth, bone, and hydroxyapatite (HA).

Composition, wt%	Enamel	Dentine	Bone	HA
Calcium	36.5	35.1	34.8	39.6
Phosphorous	17.1	16.9	15.2	18.5
Ca/P ratio	1.63	1.61	1.71	1.67
Total inorganic (%)	97	70	65	100
Total organic (%)	1.5	20	25	—
Water (%)	1.5	10	10	—

**Table 2:** Crystallographic properties: lattice parameters (±0.003 Å).

Composition, wt%	Enamel	Dentine	Bone	HA
*a*-axis (Å)	9.441	9.421	9.41	9.430
*c*-axis (Å)	6.880	6.887	6.89	6.891
Crystallinity index, (HA = 100)	70–75	33–37	33–37	100

**Table 3:** Typical properties of dense hydroxyapatite.

Properties	Amount
Theoretical density	3.156 g/cm^3^
Hardness	500–800 Vickers, 2000–3500 Knoop
Tensile strength	40–100 MPa
Bend strength	20–80 MPa
Compressive strength	100–900 MPa
Fracture toughness	1 MPam^1/2^
Young's modulus	70–120 GPa
